# Supplementary material for: The novel Dbl homology/BAR domain protein, MsgA, of Talaromyces marneffei regulates yeast morphogenesis during growth inside host cells
Source: Sci Rep. 2021 Jan 27;11:2334. doi: 10.1038/s41598-020-79593-4 (PMC7840665; doi:10.1038/s41598-020-79593-4)
Supplement: Supplementary file 1 — Supplementary Information [file 41598_2020_79593_MOESM1_ESM.pdf]

The novel *Dbl* homology/BAR domain protein, MsgA, of *Talaromyces marneffei* regulates yeast morphogenesis during growth inside host cells

Harshini Weerasinghe, Hayley E. Bugeja and Alex Andrianopoulos*

Genetics, Genomics and Systems Biology, School of BioSciences, University of Melbourne, Victoria 3010, Australia

Keywords: *Talaromyces marneffei*, *Penicillium marneffei*, macrophage infection, Rho guanine nucleotide exchange factor, yeast morphogenesis, pathogenic fungi, BAR domain.

*Corresponding author: Alex Andrianopoulos, Genetics, Genomics and Systems Biology, School of BioSciences, University of Melbourne, Victoria 3010, Australia. Telephone + 61 3 8344 5164. Email alex.a@unimelb.edu.au

Supplementary Data

Supplementary Figure 1: Protein domain composition and sequence features of the MsgA protein. The MsgA protein has a unique structural domain arrangement that differs from other canonical Rho GEF proteins. Additionally, sequence alignment of the MsgA protein reveals a region containing tandem glutamic acid repeats, showing variation in number amongst dimorphic pathogens, members of the *Talaromyces* clade and *T. marneffei* clinical isolates. (A) Schematic comparison of protein domain structure for Rho GEF proteins in *T. marneffei.* Domains are indicated as coloured boxes. Rho GEFs posses a number of domains but those principally required for function are the dbl homology domain (DH)(blue) and the Plekstrin homology domain (PH)(purple). Other domains include Phox and Bem1 (PB1)(yellow), Citron homology (CNH)(green) and CDC24/ SCD1 (aqua). The MsgA protein has a specific domain structure, which includes a DH and Bin-Amphiphysin-Rvs domain (BAR)(red) domains. The vertical black bar within the N-terminus of the protein represents the 26 glutamic acid repeat sequence. Numbers represent amino acid residues in all Rho GEF proteins. (B) Sequence alignment of the tandem repeated glutamic acid region of the MsgA protein for *Talaromyces funiculosus* (Tf), *Talaromyces flavus* (Tfl), *Talaromyces stipitatus* (Ts) and 14 isolates of *T. marneffei*. *T. marneffei* type strain (Tm 2161) and clinical isolates are separated from the *Talaramycete* species by a dashed line. While the Tf, Tfl and Ts species show the smallest number of glutamic acid repeats, each of the *T. marneffei* isolates show a varying number of repeats ranging from 15 - 26. The *T. marneffei* isolates include clinical isolates from Hong Kong (Pm1) and Thailand (F4), various isolates from AIDS patients (3482, 3840, 3041, 3871, 4059, 027 and 043) and suspected immunocompetent patients (012, 203 and 702), and isolates from the natural host of *T. marneffei*, the Bamboo rat (*Rhizomys sinensis*) (HR2 and BR2). (C) Sequence alignment of the glutamic acid region (grey) for *T. marneffei* and other non-pathogenic and pathogenic fungal species. Tandem repeats of glutamic acid are seen only in the *T. marneffei* orthologue of MsgA. The *T. marneffei* (Tm 2161) sequence used in the alignments is the type strain. Other species include non-pathogenic *Aspergillus nidulans* (An, AN3754.2), pathogenic *Aspergillus fumigatus* (Af, AFUA_7G04600) and dimorphic pathogens *Coccidioides immitis* (Ci, CIMG_07534), *Histoplasma capsulatum* (Hc, HCBG_07642) and *Paracoccidioides brasiliensis* (Pb, PADG_12531). The (*****) indicates positions which have a single, fully conserved residue, (**:**) indicates conservation between residues that have strongly similarities and (**.**) indicates conservation between residues that have weak similarities. Alignments were done using CLUSTAL O (1.2.0).

Supplementary Figure 2: The filaments produced by the Δ*msgA* strain are dissimilar to 37˚C *in vitro* arthroconidia or yeast at the transcriptional level.
Genes with known 37˚C *in vitro* specific expression (represented by PMAA gene IDs) were examined in the wildtype and Δ*msgA* strains, during both *in vitro* and intracellular macrophage growth and used to determine if the aberrant filaments observed in the Δ*msgA* mutant strain were similar to 37˚C *in vitro* growth. RNA isolated from wildtype grown for 6 days in liquid BHI medium (WT *in vitro*), ΔmsgA strain grown in LPS activated J774 murine macrophages for 24 hours *(*Δ*msgA* intracellular) and wildtype grown in LPS activated J774 murine macrophages for 24 hours (WT intracellular) was used for qPCR expression analysis. The histone H3 gene was used as a control (H3). The Δ*msgA* strain does not show inappropriate expression of these genes during macrophage infection and the expression pattern is comparable to that seen in wildtype isolated from within macrophages, suggesting that the filaments and elongated yeast cells observed during macrophage infection of this strain are not similar to 37˚C *in vitro* grown yeast.

Supplementary Figure 3: The Δ*msgA* strain produces elongated yeast cells at 24 hours post infection in THP-1 macrophages.
Differentiated THP-1 human macrophages were infected with conidia from the wildtype, Δ*msgA* and complemented Δ*msgA* $msgA^{+}$ strains and examined microscopically after 24 hours. (A) At 24 hr post infection wildtype ($msgA^{+}$) *T. marneffei* within THP-1 macrophages produced numerous ovoid yeast cells that divided by fission. In contrast the Δ*msgA* mutants show increased yeast cell length. The complemented strain (Δ*msgA* $msgA^{+}$) produced yeast cells of comparable length to wildtype. (B) The effect of deleting *msgA* on morphogenesis in macrophages was quantified at the 24 hr post infection time point. Wildtype yeast cells showed an average length of 4.9±0.92μm compared to the Δ*msgA*

mutant strain, which produced yeast cells of an average cells length of 8.6±2.5μm, approximately 1.7 times longer than wildtype. Images were captured using differential interference contrast (DIC) or with epifluorescence to observe fungal cell walls stained with calcofluor white (CAL). Error bars represent the standard error of the mean with t-test values falling in the following range *** ≤ 0.001. Scale bars are 10 μm.

Supplementary Figure 4: The *msgA* gene affects conidiation during asexual development.

The *msgA* gene is necessary to achieve wildtype levels of conidiation and its loss attenuates conidial production. (A) Wildtype (*msgA*$^+$), *ΔmsgA,* complemented (*ΔmsgAmsgA*$^+$) and *xylP(p)::msgA* strains were grown for 10 days in either unsupplemented (upper panel) or 0.5% xylose supplemented (lower panel) ANM medium. (B) When media is not supplement with xylose the *ΔmsgA* mutant strain and the *xylP(p)::msgA* over expression strain showed a reduction in conidiation compared to wildtype ($5.3x10^8$±0.4 and $7.7x10^8$±0.8 conidia/ml vs $23.9x10^8$±0.9 conidia/ml respectively). However in the presence of xylose that drives induction of the *msgA* gene in the *xylP(p)::msgA* strain, conidiation is restored to wild type levels ($35.2x10^8$±1.5 conidia/ml for the *xylP(p)::msgA* strain compared to $33x10^8$±1.5 conidia/ml in the wild type). This phenotype was not completely rescued in the complementation strain ($15.9x10^8$±1.1 conidia/ml in ANM and $18.1x10^8$±1.4 conidia/ml in ANM+ 0.5% xylose). Error bars represent standard error of the mean with t-test values falling in the following range ** ≤ 0.05 and *** ≤ 0.001.

Supplementary Table 1: Primers used in this study.

Primer ID	Primer sequences	Origin
VV55	attgccatagagaccgct	This study
VV56	tgtagaactcccccaggt	This study
VV57	ggggacccagctttcttgtacaaagtggtaaggagacagcatagcac	This study
VV58	ggggagcctgcttttttgtacaaacttgtgaaggactgaagcggtta	This study
WW42	aggatgtaaccgttgagg	This study
WW44	caacaaaaactgggacctatt	This study
WW45	tgtgacctacgatgattagca	This study
WW46	tttttctagaaccaagctttgcaaaatgctgt	This study
WW57	caattcactggccgtcgtttt	This study
WW59	gtggtcggtcaagttgttagc	This study
WW60	ctgttctggtgttggtgagcc	This study
WW77	ccgttggcgaggatggtgata	This study
WW78	agaaacaaacctggctaccct	This study
WW79	aaaccaccgaagtctatccaa	This study
H57	tgtaaaacgacggccagt	(Boyce et al., 2010)
H56	ggaaacagctatgaccatg	(Boyce et al., 2010)

Supplementary Table 2. *T. marneffei* strains used in this study.

Strain ID	Full Genotype	Origin
G809	*ΔligD::pyrG*$^{+}$*niaD pyrG*	Bugeja *et al.*, 2012
G816	*ΔligD niaD*1 *pyrG*	Bugeja *et al.*, 2012
G829	*ΔligD ΔriboB::pyrG*$^{+}$ *niaD pyrG*	Bugeja *et al.*, 2012
G1044	*ΔligD niaD pyrG ΔmsgA::pyrG*$^{+}$	This study
G1045	*ΔligD niaD pyrG ΔmsgA::pyrG*$^{+}$ [niaD^{t} *msgA*$^{+}$]	This study
G1046	*ΔligD niaD pyrG ΔmsgA::pyrG*$^{+}$ [niaD^{t} *msgA*$^{+}$::mCherry]	This study
G1047	*ΔligD niaD pyrG ΔmsgA::pyrG*$^{+}$ [niaD^{t} *msgA*$^{\Delta BAR}$]	This study
G1048	*ΔligD niaD pyrG ΔmsgA::pyrG*$^{+}$ [niaD^{t} *msgA*$^{\Delta DH}$]	This study
G1049	*ΔligD niaD pyrG ΔmsgA::pyrG*$^{+}$ [niaD^{t} *msgA*$^{\Delta BAR}$*::mCherry*]	This study
G1050	*ΔligD niaD pyrG ΔmsgA::pyrG*$^{+}$ [niaD^{t} *msgA*$^{\Delta DH}$*::mCherry*]	This study
G1051	*ΔligD niaD pyrG*$^{+}$ *xylP(p)::msgA::bar*$^{+}$	This study

Supplementary Table 3. Kinetics of conidial germination during *in vitro* growth and macrophage infection.

In vitro			
**Strain**	**16 hours**	**24 hours**	**48 hours**
msgA⁺	59±2.47	89.71±3.06	98.1±2.62
Δ*msgA*	53.8±0.49	86.71±1.21	96.04±0.76
**Intracellular growth**			
**Strain**	**4 hours**	**6 hours**	
msgA⁺	38.8±3.5	72.7±9.5	
Δ*msgA*	37.1±2	75.3±5	

A

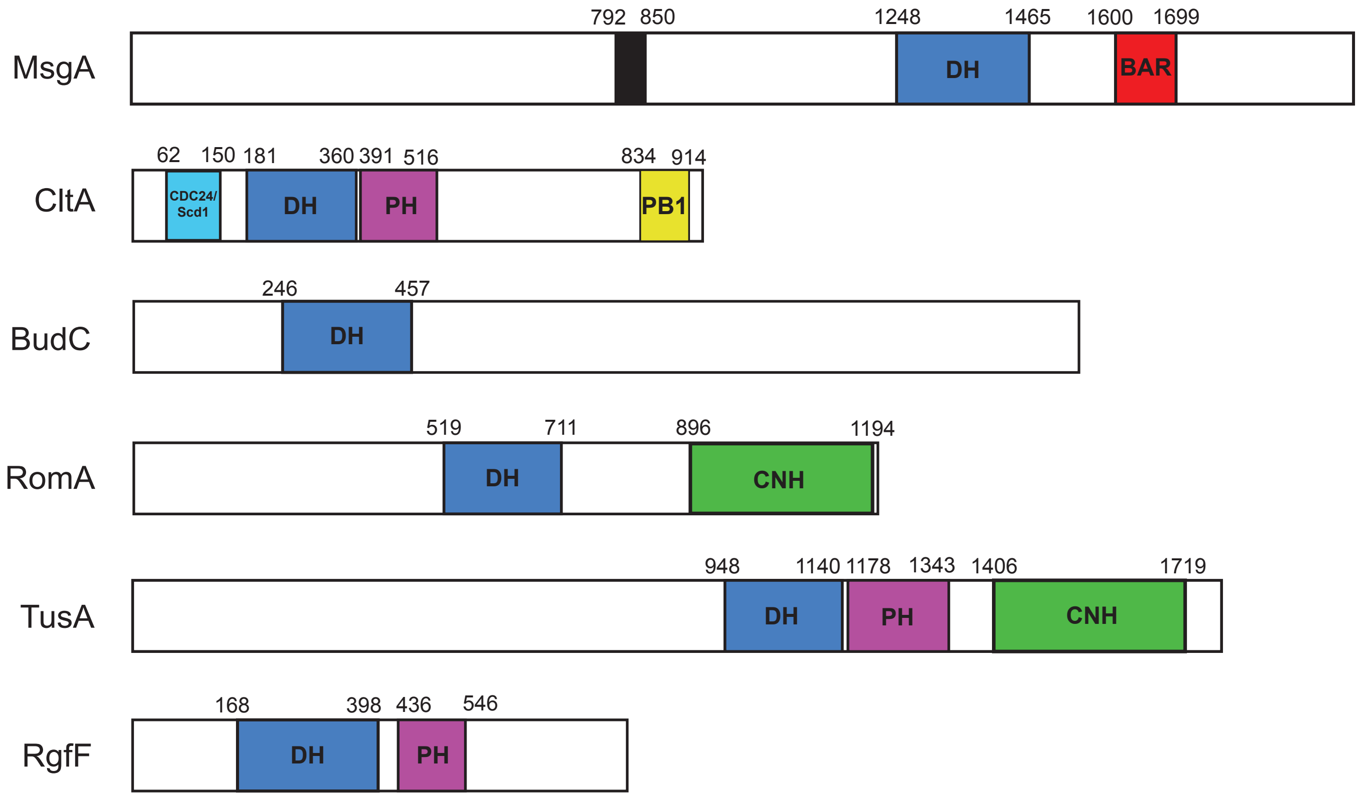

B

```
Tf        769    ASHRDLLSRIMQLRESSSSSECDEEEED----------------------------DDDD
Tfl       788    ASHRDLLSRIMQLRESSSSSECDEEEED----------------------------DDDN
Ts        788    ESHRDLLSRIMQLRESSSSSECDEDED-----------------------------DDDD
- - - - - - - - - - - - - - - - - - - - - - - - - - - - - - - - - - - - - - -
Tm 2161   780    ASHRDLLSRIMQLRESSSSSECDEEEEEEEEEEEEEEEEEEEEEEEEEEDDDDDDEDEDD
Pm1       780    ASHRDLLSRIMQLRESSSSSECDEEEEEEEEE---------EEEEEEEEEEDDDDDDEDD
F4        780    ASHRDLLSRIMQLRESSSSSECDEEEEEEEEE----------EEEEEEEDDDDDDEDEDD
3482      780    ASHRDLLSRIMQLRESSSSSECDEEEEEEEEE-----------EEEEEEEEDDDDDDEDD
3840      727    ASHRDLLSRIMQLRESSSSSECDEEEEEEEEE---------EEEEEEEEDDDDDDEDEDD
3841      780    ASHRDLLSRIMQLRESSSSSECDEEEEEEEEEEEE---EEEEEEEEEEDDDDDDDEDEDD
3871      780    ASHRDLLSRIMQLRESSSSSECDEEEEEEEEE-----------EEEEEEEEDDDDDDEDD
4059      780    ASHRDLLSRIMQLRESSSSSECDEEEEEEEEE---------EEEEEEEEDDDDDDEDEDD
027       780    ASHRDLLSRIMQLRESSSSSECDEEEEEEEEE-----------EEEEEDDDDDDDEDEDD
043       780    ASHRDLLSRIMQLRESSSSSECDEEEEEEEEE----------EEEEEEDDDDDDDEDEDD
012       780    ASHRDLLSRIMQLRESSSSSECDEEEEEEEEE-------------EEEDDDDDDDEDEDD
203       780    ASHRDLLSRIMQLRESSSSSECDEEEEEEEEE-----------EEEEEDDDDDDDEDEDD
702       780    ASHRDLLSRIMQLRESSSSSECDEEEEEEEEE-----------EEEEEEDDDDDDEDEDD
HR2       780    ASHRDLLSRIMQLRESSSSSECDEEEEEEEEE-------------EEEEEEDDDDDDEDD
BR2       780    ASHRDLLSRIMQLRESSSSSECDEEEEEEEEE-----------EEEEEDDDDDDDEDEDD
                  ***********************:*:                            *:*:
```

C

```
Tm 2161   780    ASHRDLLSRIMQLRESS-SSSECDEEEEEEEEEEEEEEEEEEEEEEEEEEEDDDDDDEDEDDNELCDDEND
An        753    TSHRTLLNEIMRIRESSPSSSSCDEQE------------------------------------YGFSDND
Af        758    ASHRTLLSQIMQIRESSPDTSSCDEAD------------------------------------YSFSDTE
Ci        722    QSHRSMLSHIMQMRESSPDITDSSDDDE--------------------------------DTSSSGR---
Hc        760    QIHRTVLSQIMQMRESSPSSSSSSD---------------------------------------SFSERD
Pb        761    QIHRTVLSQIMQMRESSPSSSAYSD---------------------------------------SFSERD
                   ** .:  ** .****
```

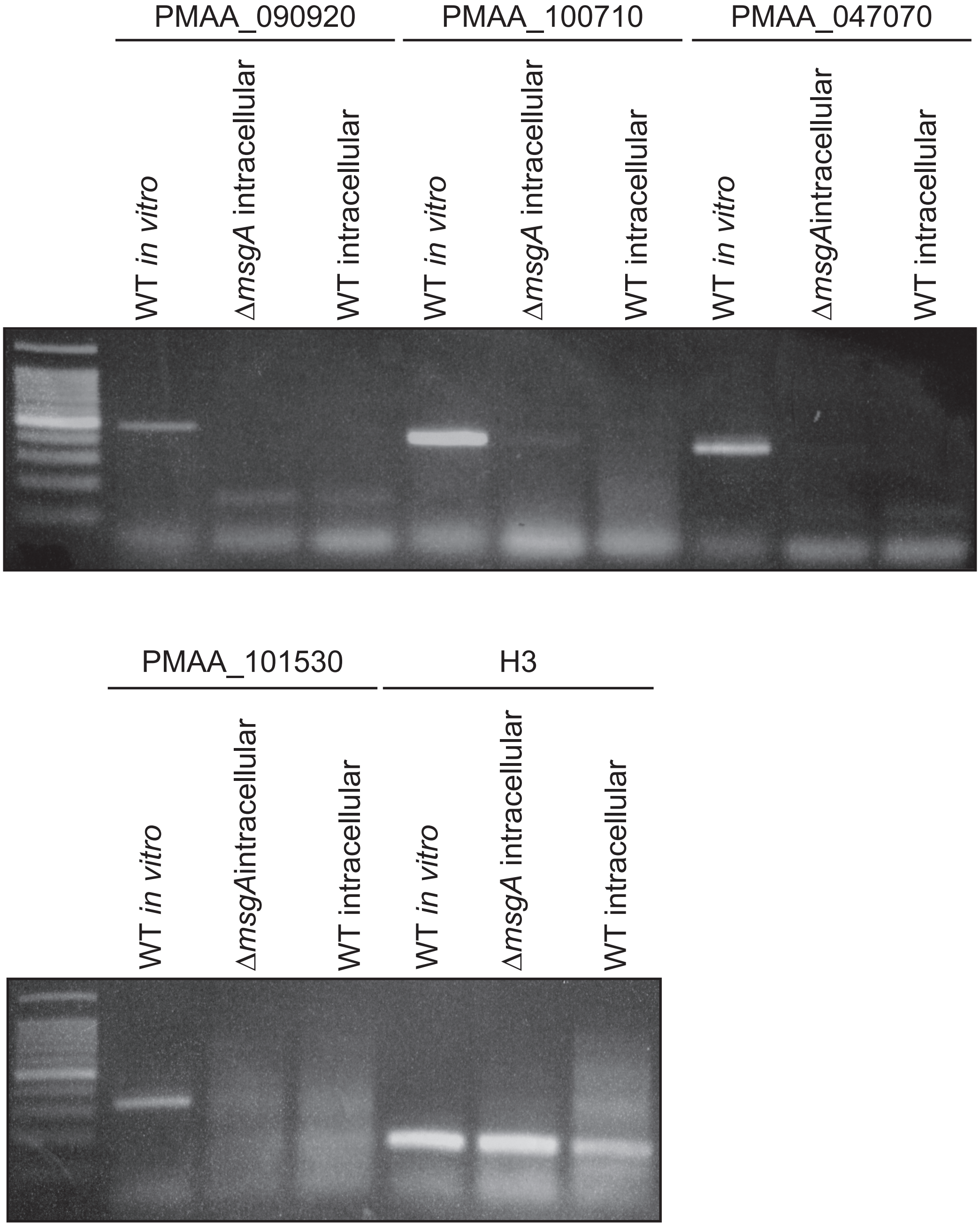
PMAA_090920
PMAA_100710
PMAA_047070
WT *in vitro*
Δ*msgA* intracellular
WT intracellular
WT *in vitro*
Δ*msgA* intracellular
WT intracellular
WT *in vitro*
Δ*msgA*intracellular
WT intracellular
PMAA_101530
H3
WT *in vitro*
Δ*msgA*intracellular
WT intracellular
WT *in vitro*
Δ*msgA* intracellular
WT intracellular

A

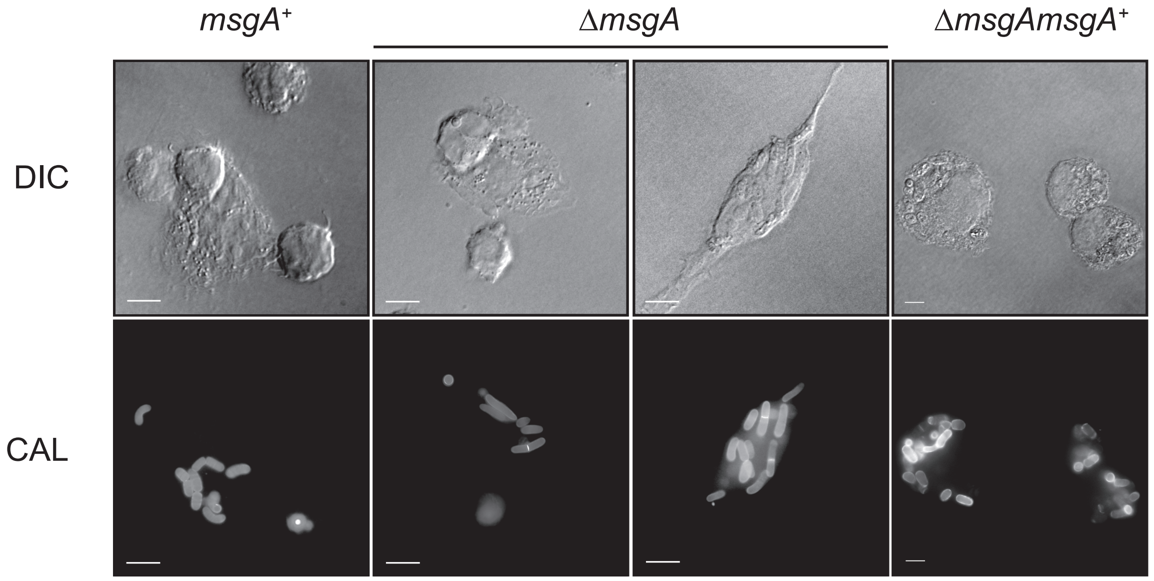

msgA+
ΔmsgA
ΔmsgAmsgA+
DIC
CAL

B

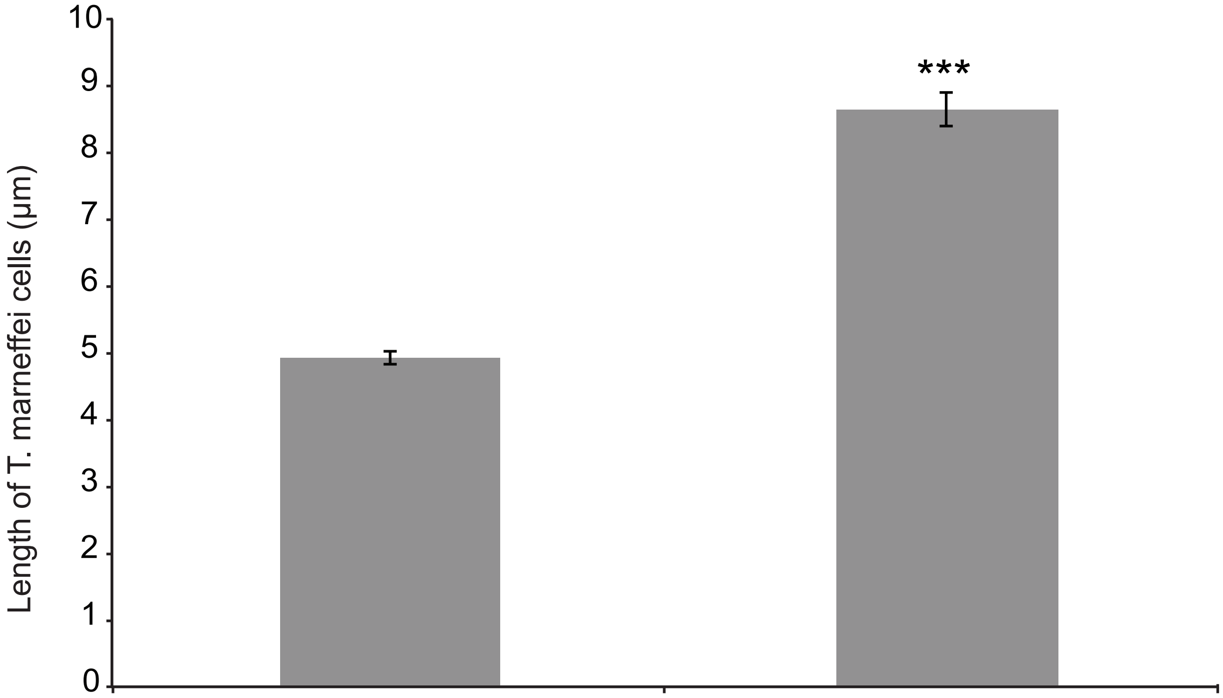

10
9
8
7
6
5
4
3
2
1
0
Length of T. marneffei cells (μm)
***
msgA+
ΔmsgA

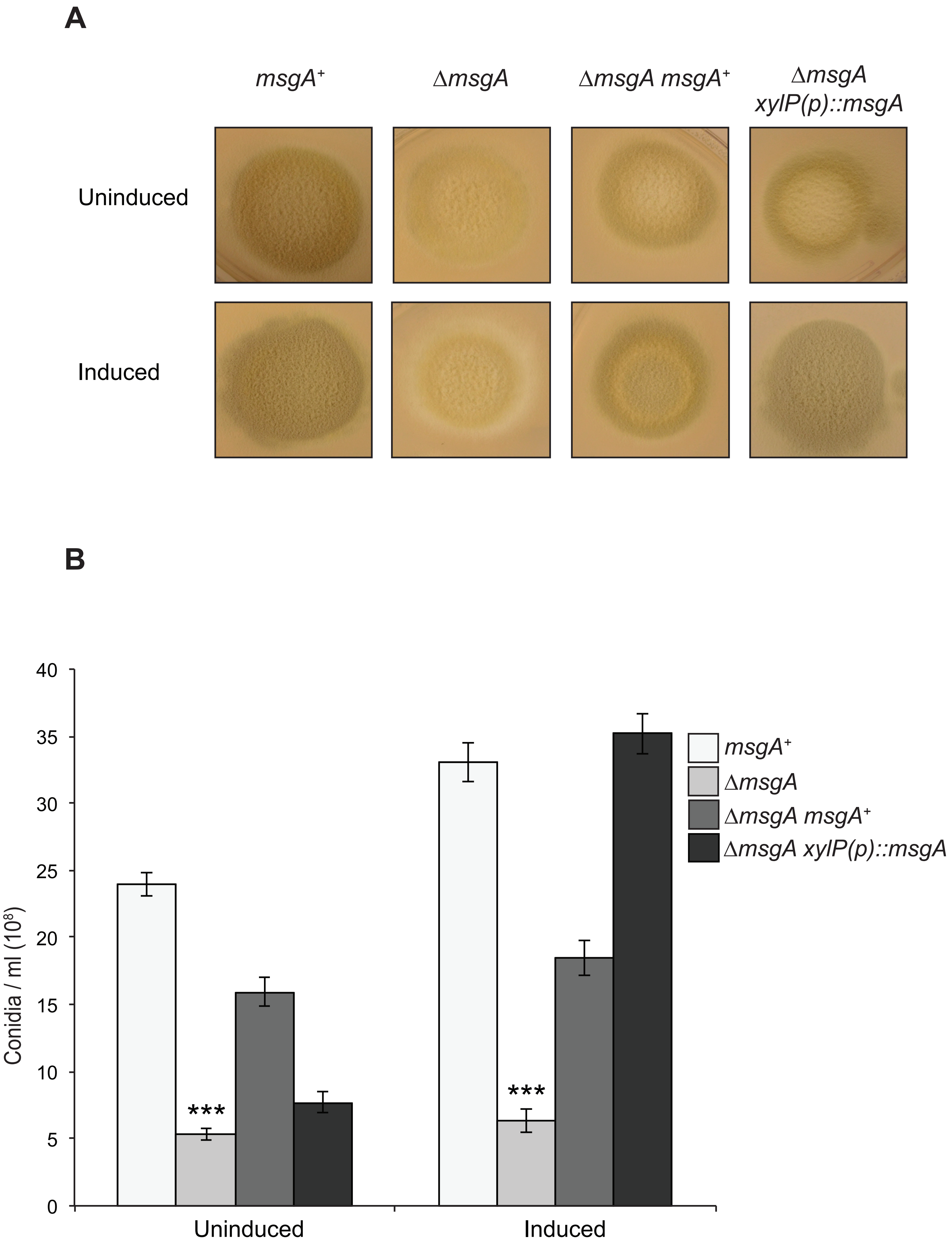
A
msgA⁺
ΔmsgA
ΔmsgA msgA⁺
ΔmsgA xylP(p)::msgA
Uninduced
Induced
B
Conidia / ml (10⁸)
40
35
30
25
20
15
10
5
0
***
***
Uninduced
Induced
msgA⁺
ΔmsgA
ΔmsgA msgA⁺
ΔmsgA xylP(p)::msgA
